# Ultrasound assessment of the infrapatellar fat pad can detect Hoffa-synovitis in patients following anterior cruciate ligament reconstruction: A pilot study^☆^

**DOI:** 10.1016/j.ostima.2024.100174

**Published:** 2024-02-01

**Authors:** M Fagan, R Fajardo, C Grozier, TR Jildeh, M Lissy, MS Harkey

**Affiliations:** a College of Health Professions, Grand Valley State University, USA; b Lansing Radiology Associates, USA; c Department of Kinesiology, Michigan State University, USA; d Michigan State University Sports Medicine, USA

**Keywords:** IPFP, ACLR, MRI, ACLOAS

## Abstract

**Introduction:**

Osteoarthritis (OA) commonly occurs following anterior cruciate ligament reconstruction (ACLR), affecting over 50 % of patients within 10–15 years post-ACLR. The Hoffa-synovitis of the infrapatellar fat pad (IPFP) has been implicated as a major contributor to OA pathogenesis. While MRI is typically used to evaluate the IPFP, it is cost-prohibitive for routine screening. This study aimed to validate ultrasound as an alternative for detecting IPFP Hoffa-synovitis in participants post-ACLR.

**Methods:**

In this cross-sectional study, 15 participants (18–35 years, 1–5 years post-ACLR) underwent two imaging sessions separated by one week. First, a standardized bilateral anterior knee ultrasound assessment was used to examine IPFP echo-intensity. Second, MRI scans of both knees were graded by a board-certified musculoskeletal radiologist for Hoffa-synovitis according to the Anterior Cruciate Ligament Osteoarthritis Score grading system. IPFP echo-intensity were quantified on each ultrasound image, and a limb symmetry index (LSI) was calculated to assess between-limb differences. We used an independent *t*-test and Cohen's *d* effect sizes to compare IPFP echo-intensity LSI between people with and without MRI-confirmed Hoffa-synovitis.

**Results:**

Four of the 15 participants (27 %) exhibited MRI-confirmed Hoffa-synovitis. Significantly higher IPFP echo-intensity LSI values were found in participants with Hoffa-synovitis (32.1 ± 12.1 %) compared to those without (10.5 ± 10.4 %), confirming the ultrasound's ability to distinguish between the two groups (*t* = -3.44; *p* = 0.004; *d* = 2.01).

**Discussion:**

Ultrasound detects bilateral IPFP signal intensity alterations in participants post-ACLR with MRI-confirmed Hoffa-synovitis. This work should be seen as a proof-of-concept, and further validation in a larger, more diverse sample is essential for verifying these results.

## Introduction

Osteoarthritis (OA) is a common following anterior cruciate ligament injury. Current evidence indicates that, even after surgical reconstruction (ACLR), more than one-third of individuals develop radiographic OA within 10 years post-ACLR [1]. The occurrence of post-traumatic OA is linked to significant pain, functional impairments, and a diminished quality of life, highlighting the necessity for research into contributing factors [2]. A key structure implicated in early OA pathogenesis is the infrapatellar fat pad (IPFP) [3]. The IPFP serves a critical metabolic and biomechanical role within the anterior knee and is a source of inflammatory mediators such as cytokines and adipokines, especially when in a pathological state known as Hoffa-synovitis, characterized by edema, fibrosis, and macrophage infiltration [3].

Magnetic resonance imaging (MRI) is typically used to assess the IPFP and its relationship to OA development, particularly following ACLR [4], [5], [6], [7]. Early MRI measures have identified alterations in IPFP signal intensity as precursors to radiographic OA, with significant associations observed within as few as four years [4,5]. The observed signal alterations are not merely incidental; they demonstrate correlations with increased concentrations of cytokine biomarkers and cartilage degradation markers in synovial fluid samples collected prior to ACLR [6]. Recent studies have further established that these IPFP signal changes, specifically those indicative of Hoffa-synovitis, are independently correlated with cartilage damage and subsequent OA progression post-ACLR [5]. These alterations often manifest as edema and other signal irregularities, serving as markers for underlying pathological conditions that could potentially accelerate joint degeneration [4]. However, MRI's high cost, limited accessibility, and time-consuming nature present challenges for its widespread use as a monitoring tool. These limitations underline the need for developing more clinically accessible and cost-effective alternatives for assessing the IPFP in the context of OA secondary prevention strategies.

Ultrasound is a lower-cost, portable, and clinically accessible modality for musculoskeletal imaging, presenting itself as a promising alternative to MRI for assessing the IPFP [8,9]. Prior studies have successfully employed ultrasound to quantify IPFP morphology and echo-intensity in uninjured individuals [8,9]. Moreover, preliminary investigations have explored the use of ultrasound in post-ACLR patients to examine the relationship between the flexibility or thickness of the IPFP and anterior knee pain [10]. However, there is a significant gap in the literature regarding the ultrasound for detecting signal intensity alterations indicative of Hoffa-synovitis in individuals following ACLR. The need to advance and validate ultrasound methods for assessing IPFP signal intensity is clear, particularly for detecting early OA in post-ACLR patients, given MRI's cost and accessibility issues.

Currently, research on using ultrasound to detect Hoffa-synovitis in post-ACLR patients is lacking, despite its known prevalence and links to poor outcomes and OA progression. Therefore, the primary objective of this study is to evaluate the utility of ultrasound in identifying Hoffa-synovitis, as confirmed by MRI, among individuals post-ACLR. Specifically, we aim to determine if the limb symmetry index (LSI) for ultrasound-assessed IPFP signal intensity provides a within-person measure to compare affected and unaffected knees. We hypothesize that this LSI will reveal significant differences in IPFP ultrasound echo-intensity between patients with and without MRI-confirmed Hoffa-synovitis, with greater echo-intensity anticipated in the limb with the worse Hoffa-synovitis.

## Methods

### Study design

In this cross-sectional study, participants underwent two data collection sessions separated by one week. The first session included a bilateral anterior knee ultrasound examination of the IPFP, while the second session included a bilateral knee MRI assessment. Both data collection sessions were conducted within a two-hour timeframe on their respective days.

### Participants

Fifteen participants who were 1–5 years post-primary, unilateral ACLR were recruited for this study. We identified patients eligible for this study by contacting individuals that had previously participated in research studies in our laboratory. These patients were previously recruited to participate in our laboratory's research studies by recommendation from their orthopaedic surgeon. The age range of the participants was 18–35 years, and they had no history of additional contralateral or ipsilateral lower extremity surgery or osteoarthritis on radiographs that were collected as part of clinical care prior to surgery. A single board-certified musculoskeletal radiologist (RF) graded the radiographs for the absence of osteoarthritis (i.e., Kellgren-Lawrence grade < 2). This age range was chosen in accordance with the recommendations for clinical trials aimed at preventing OA in patients after ACLR [11]. Participants were not excluded based on body mass index. All participants provided written informed consent prior to enrollment in the study, which was approved by the Institutional Review Board of the local University.

### MRI grading of Hoffa-synovitis

All participants underwent MRI scanning of their ACLR and contralateral limb using the same 3T GE Excite MR Imaging scanner (GE Healthcare, Milwaukee, WI, USA) and an 8-channel phased array knee coil. The imaging protocol consisted of an intermediate-weighted fat suppressed MRI sequence. The degree of Hoffa-synovitis was assessed bilaterally by a single board-certified musculoskeletal radiologist (RF) according to the Anterior Cruciate Ligament Osteoarthritis Score grading system on a scale of 0–3 (0 = normal appearance, 1 = mild hyperintensity signal intensity, 2 = moderate hyperintensity signal intensity, 3 = severe hyperintensity signal intensity) [12]. In this study, the index limb was identified based on the worst MRI-defined Hoffa-synovitis grade, which in one case was the non-ACLR limb. This approach allows for a more comprehensive assessment of Hoffa-synovitis, irrespective of the ACLR status.

### Anterior knee longitudinal ultrasound acquisition

**Fig. 1:**
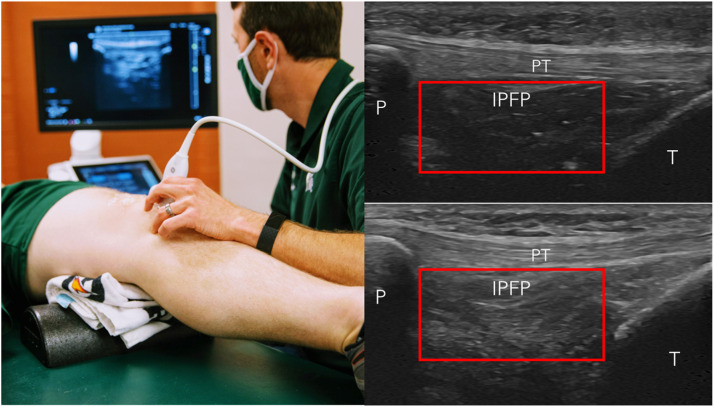
Anterior Knee Ultrasound Image Acquisition and Quantitative Assessment of Infrapatellar Fatpad Echo-intensity. Left Pane = patient and probe positioning to acquire images of the anterior knee. Right Panes = ultrasound image acquired in this patient and probe position to visualize the infrapatellar fatpad (IPFP) deep to the patellar tendon (PT), distal to the patella (P), and proximal to the tibia (T). Right top picture shows an example image of a fatpad without MRI-confirmed Hoffa-synovitis and the Right Bottom picture shows the same patient's contralateral knee with MRI-confirmed Hoffa-synovitis. The red rectangle in both pictures represents the borders of the region of interest used to quantify the IPFP signal intensity. The region of interest rectangle is standardized at a height of 10 millimeters, but the width was adjusted to fit each participant's knee as the largest width that fit within the IPFP while not including the overlying patellar tendon, the proximal patella, the distal tibia.

The ultrasound assessment was conducted by a single investigator (MH) with nine years of experience in musculoskeletal ultrasound research, using a GE LOGIQ P9 R3 ultrasound system and an L3–12-RS wideband linear array probe (GE Healthcare, Chicago, IL). To ensure consistency, all ultrasound settings were standardized across participants. The following settings were used: a depth of 4 cm, a gain of 50, an image resolution of 212 pixels per 1 cm, and a probe width of 5.5 cm. The participants were positioned supine with the knee resting on a bolster in 20–30° flexion (Fig. 1). The IPFP was assessed with longitudinal probe orientation in line with the midline of the patellar tendon at the infrapatellar attachment site (Fig. 1). Two images were recorded with the ultrasound probe being removed and repositioned prior to acquiring each image. The acquisition of the ultrasound images of the IPFP took less than one minute per knee.

### Quantifying IPFP echo-intensity LSI

A single reader (MF) used ImageJ software (https://imagej.nih.gov/) to place a standardized rectangle region of interest within the IPFP to assess the mean echo-intensity. We are using the standardized rectangle region of interest due to its ease and time efficiency (i.e., less than one minute per knee to process each image). The region of interest rectangle is standardized at a height of 10 millimeters, but the width was adjusted to fit each participant's knee as the largest width that fit within the IPFP while not including the overlying patellar tendon, the proximal patella, the distal tibia (Fig. 1). The IPFP echo-intensity was then quantified as the mean grey-scale pixel intensity ranging from 0 (black) to 255 (white) within the rectangular region of interest. We then normalized the IPFP echo-intensity between limbs by creating an LSI to quantify the percent difference between the index limb with the worst MRI-confirmed Hoffa-synovitis to the contralateral limb using the following equation:

IPFP echo-intensity LSI = [(index limb IPFP echo-intensity) – (contralateral limb IPFP echo-intensity) / (between limb average IPFP echo-intensity)]*100.

For limbs with no MRI-confirmed Hoffa-synovitis, the numerator in the LSI was calculated as (ACLR limb IPFP echo-intensity – contralateral limb IPFP echo-intensity). For the one participant who had MRI-confirmed Hoffa-synovitis in both limbs, the numerator in the LSI was calculated as (worst MRI grade limb IPFP echo-intensity – best MRI grade limb IPFP echo-intensity). A IPFP echo-intensity LSI value greater than 0 % indicates a brighter IPFP signal intensity compared to the contralateral limb.

### Statistical analysis

Means and standard deviations or frequency and percentages were calculated for common demographic variables and the IPFP echo-intensity variables. We used independent *t*-tests to compare common mean demographics (i.e., body mass index, age, months post-ACLR) between participants with and without MRI-confirmed Hoffa-synovitis. We used chi-square tests to compare frequency of categorical variables (i.e., sex, graft type) between participants with and without MRI-confirmed Hoffa-synovitis. A Shapiro-Wilk test was used to determine that IPFP echo-intensity LSI was normally distributed. We used an independent *t*-test to compare the IPFP echo-intensity LSI, affected limb IPFP echo-intensity, and contralateral limb IPFP echo-intensity between individuals with and without Hoffa-synovitis. Additionally, we calculated Cohen's *d* effect sizes and mean differences with 95 % CI to describe the magnitude of the difference in IPFP echo-intensity metrics between the two groups [13]. Using previously established cut thresholds, we categorized effect sizes as small = 0.20–0.49, medium = 0.50–0.79, or large = 0.80 [13]. All statistical analyses were performed using Jamovi software (version 1.8) with an *a priori* α level of 0.05.

## Results

**Fig. 2:**
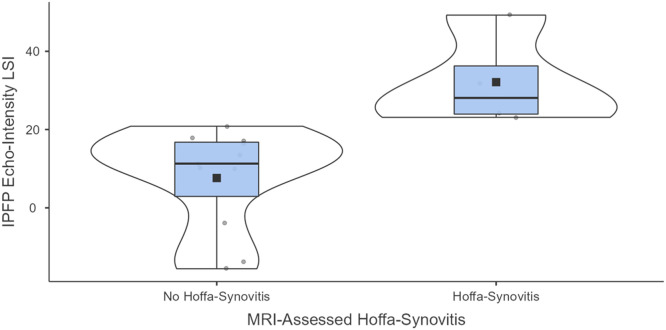
Differences in IPFP Echo-intensity LSI between participants with and without MRI-confirmed Hoffa-synovitis.

Table 1 highlights the demographics for all participants, as well as for participants with and without MRI-confirmed Hoffa-synovitis. Of the 15 participants, 4 individuals presented with Hoffa-synovitis in their ACLR limb and 1 of these participants also had Hoffa-synovitis in their contralateral limb. The IPFP echo-intensity LSI in participants with Hoffa-synovitis (mean±standard deviation: 32.1 ± 12.1 %) was significantly higher (*t* = −3.44; *p* = 0.004; *d* = 2.01, 95 % CI = −3.36, −0.61; mean difference = −21.7 %, 95 % CI = −35.3, −8.1) when compared to participants without Hoffa-synovitis (10.5 ± 10.4 %) (Table 1, Fig. 2). Raw IPFP echo-intensity in the affected or contralateral limbs was not significantly different between participants with and without MRI-detect Hoffa-synovitis (Table 1).

**Table 1:** Physical Characteristics of Participants.

	All (*n* = 15)	No Hoffa-Synovitis (*n* = 11)	Hoffa-Synovitis (*n* = 4)	t (p-value)* χ^2^ (p-value)^	Mean Difference (95 % CI)
**Body Mass Index (kg/m^2^)**	23.5 ± 3.2	23.3 ± 3.0	24.0 ± 4.0	−0.34 (0.74)*	−0.6 (−4.8, 3.5)
**Age (years)**	25.8 ± 5.8	25.7 ± 5.8	26.0 ± 6.5	−0.08 (0.94)*	−0.2 (−7.8, 7.3)
**Months Post ACLR**	29.3 ± 12.7	32.0 ± 12.9	21.8 ± 10.1	1.42 (0.18)*	10.3 (−5.3, 25.8)
**Sex [n(%) female]**	9 (60 %)	7 (64 %)	2 (50 %)	0.23 (0.63)^	
**Graft type**				1.15 (0.56)^	
*Hamstring Tendon*	8 (53 %)	5 (45.5 %)	3 (75 %)		
*Patellar Tendon*	6 (40 %)	5 (45.5 %)	1 (25 %)		
*Quad Tendon*	1 (7 %)	1 (9 %)	0 (0 %)		
**ACLR Hoffa-Synovitis Grade**				15.0 (<0.001)^	
*Grade 0*	11 (73 %)	11 (100 %)	0 (0 %)		
*Grade 1*	4 (27 %)	0 (0 %)	4 (100 %)		
*Grade 2*	0 (0 %)	0 (0 %)	0 (0 %)		
*Grade 3*	0 (0 %)	0 (0 %)	0 (0 %)		
**Contralateral Hoffa-Synovitis Grade**				2.95 (0.09)^	
*Grade 0*	14 (93 %)	11 (100 %)	3 (75 %)		
*Grade 1*	0 (0 %)	0 (0 %)	0 (0 %)		
*Grade 2*	1 (7 %)	0 (0 %)	1 (25 %)		
*Grade 3*	0 (0 %)	0 (0 %)	0 (0 %)		
**IPFP echo-intensity**					
*Index Limb*	57.8 ± 9.0	57.2 ± 8.9	59.2 ± 10.7	−0.38 (0.71)*	−2.0 (−13.8, 9.7)
*Contralateral Limb*	51.9 ± 9.7	53.0 ± 8.8	48.7 ± 12.5	0.76 (0.46)*	4.3 (−8.0, 16,7)
*Limb Symmetry Index*	16.2 ± 14.4	10.5 ± 10.4	32.1 ± 12.1	−3.44 (0.004)*	−21.7 (−35.3, −8.1)

## Discussion

The present pilot study sought to evaluate the utility of ultrasound in identifying Hoffa-synovitis as compared to the gold standard MRI among individuals within 1–5 years post-ACLR. We found that 27 % (4 out of 15) of the participants presented with Hoffa-synovitis in the ACLR limb. The primary finding of this pilot study was that IPFP echo-intensity LSI was significantly greater in participants with Hoffa-synovitis compared to those without. The preliminary finding of this pilot study implies that an ultrasound assessment of the IPFP echo-intensity corroborates an MRI assessment of Hoffa-synovitis and may represent a clinically accessible tool for differentiating between patients with pathological IPFP changes.

The importance of the IPFP following ACLR is likely linked to the dysregulated inflammatory response following ACLR that contributes to cartilage degradation and cartilage breakdown biomarkers within the knee joint [14], [15], [16]. Inflammatory cytokine levels (e.g., IL-6, IL-8, IL-10, TNF-α, and IFN-γ) in the synovial fluid are not only increased, but also correlate with IPFP fibrosis and have been associated with poorer clinical outcomes (e.g., Lysholm score) [6]. A heightened inflammatory environment has systemic implications, affecting both cartilage and muscular changes [14], and may exacerbate OA progression and long-term pain [5]. Additionally, IPFP volume increases from 6 to 12 months post-ACLR and the amount of change is associated with patient-reported knee function at 12 months [7]. Given these interconnected factors, the use of ultrasound, a cost-effective and clinically viable imaging modality, is vital for longitudinal monitoring of IPFP changes indicative of inflammation in individuals post-ACLR.

While prior MRI-based research has underscored the role of the IPFP in OA progression, these studies often employ subjective assessments of the IPFP [17]. However, recent work have validated a quantitative evaluation of IPFP signal intensity as a more granular IPFP assessment. Specifically, prior studies have highlighted that IPFP signal intensity alterations are associated with incident radiographic OA development [18], OA progression [4], and future knee replacement [19]. Additionally, baseline IPFP signal intensity is predictive of knee symptoms and structural changes in older adults [20], indicating the broader clinical utility of these quantitative metrics. To our knowledge, our pilot study is the first to assess IPFP ultrasound echo-intensity as a cost-effective and clinically accessible alternative to MRI to assess IPFP signal intensity alterations. Our findings reveal a significant difference in IPFP echo-intensity LSI between participants with and without Hoffa-synovitis. The early detection of synovitis and changes in IPFP signal intensity could serve as an easily identifiable marker for impending morphological joint alterations, thus allowing for timely, targeted interventions. These could range from anti-inflammatory medications to complement rehabilitation strategies (e.g., strengthening, physical activity promotion) designed to prevent the onset and mitigate the progression of OA. Additionally, the non-invasive nature and ubiquity of ultrasound technology make it a feasible option for ongoing, regular monitoring of the IPFP and other pathological IPFP changes, although fully powered longitudinal studies are required.

The present study is not without limitations. While our findings demonstrate promising results in using IPFP LSI to distinguish knees with and without MRI-confirmed Hoffa-synovitis, we acknowledge the small sample size (*n* = 4 with Hoffa-synovitis) limits the impact of our pilot study. The absence of a formal power analysis and the small sample size, determined by participant availability, highlight the preliminary nature of our results. This work should be seen as a proof-of-concept, and further validation in a larger, more diverse sample is essential for verifying these results. Additionally, our focus on the early stages post-ACLR may not capture the complexities or risks associated with more advanced OA conditions. Thus, long-term studies are imperative to determine whether higher IPFP echo-intensity LSI are indicative of a more rapid trajectory towards OA onset and progression. Future investigations should also explore more refined ultrasound methodologies—such as transducer type, scanning planes, and specific IPFP boundary delineations—to improve diagnostic accuracy. While our study focused on the IPFP LSI measure, the utility of an LSI measure in a broader OA population merits consideration. We defined an index limb based on the MRI-defined presence of Hoffa-synovitis, however, this operational definition will not be possible in all contexts. For example, OA patients with bilateral OA, it may be unclear which knee to define as their index knee. Additionally, none of our participants presented with symmetrical MRI-defined Hoffa-synovitis and it is unclear how to apply our IPFP LSI measure if an individual presented with symmetric grades in both limbs. Future research should explore the adaptability of IPFP LSI in varied clinical situations, including those without clear unilateral injury markers.

In summary, our pilot study underscores the promise of using quantitative ultrasound as a tool for identifying early signal intensity asymmetries in the IPFP of participants post-ACLR. The IPFP echo-intensity LSI enables a within-person echo-intensity comparison, thereby isolating localized pathological alterations. These preliminary findings hold substantive implications as a portable, cost-effective ultrasound technique to assess the IPFP in patients at risk for OA development.

### Declaration of generative AI and AI-Assisted technologies in the writing process

During the preparation of this manuscript, the authors utilized ChatGPT, a generative AI technology, for assisting in the drafting and refining of the manuscript. ChatGPT was used for guidance on stylistic and grammatical aspects, thereby improving the overall quality of writing. Following the use of ChatGPT, the authors conducted a comprehensive review and revision of the AI-generated content. This ensured that the final manuscript accurately represents the author's original research and insights. The author take full responsibility for the content of this publication, upholding the standards of ethical research and writing practices.
